# Statistical Properties of Multivariate Distance Matrix Regression for High-Dimensional Data Analysis

**DOI:** 10.3389/fgene.2012.00190

**Published:** 2012-09-27

**Authors:** Matthew A. Zapala, Nicholas J. Schork

**Affiliations:** ^1^Department of Radiology, University of California at San DiegoLa Jolla, CA, USA; ^2^Department of Molecular and Experimental Medicine, The Scripps Translational Science Institute, The Scripps Research InstituteLa Jolla, CA, USA

**Keywords:** regression analysis, multivariate analysis, distance matrix, simulation

## Abstract

Multivariate distance matrix regression (MDMR) analysis is a statistical technique that allows researchers to relate *P* variables to an additional *M* factors collected on *N* individuals, where *P* ≫ *N*. The technique can be applied to a number of research settings involving high-dimensional data types such as DNA sequence data, gene expression microarray data, and imaging data. MDMR analysis involves computing the distance between all pairs of individuals with respect to *P* variables of interest and constructing an *N* × *N* matrix whose elements reflect these distances. Permutation tests can be used to test linear hypotheses that consider whether or not the *M* additional factors collected on the individuals can explain variation in the observed distances between and among the *N* individuals as reflected in the matrix. Despite its appeal and utility, properties of the statistics used in MDMR analysis have not been explored in detail. In this paper we consider the level accuracy and power of MDMR analysis assuming different distance measures and analysis settings. We also describe the utility of MDMR analysis in assessing hypotheses about the appropriate number of clusters arising from a cluster analysis.

## Introduction

Contemporary biological research has become increasingly data and information intensive. Technologies such as high-throughput DNA sequencing and genotyping platforms, gene expression microarrays, imaging technologies, and continuous clinical monitoring devices provide researchers with an unprecedented amount of data for individual investigations. As a result, appropriate multivariate data analysis methods are necessary in order to test specific hypotheses or extract meaningful patterns from the data generated by these technologies. Unfortunately, many traditional data analysis procedures are not immediately applicable to high-dimensional data sets. The reasons for this are somewhat obvious in that most traditional statistical methods were designed to test very specific hypotheses in settings for which the sample size, *N*, is much greater than the number of variables, *P*, collected on the individuals used to test the relevant hypotheses (i.e., *N* ≫ *P*; Donoho, 2000; Johnstone and Titterington, 2009).

DNA sequencing, microarray, imaging, and related studies typically generate huge amounts of data that, due to their expense and sophistication, are often collected on a relatively small number of individuals. Thus, it is typically the case that *P* ( *N* in these studies. In these settings, standard univariate data analysis strategies that focus on a specific hypothesis test involving each variable are inappropriate, and their naïve application could potentially generate an enormous number of false positive findings. As an alternative to classical univariate procedures – as well as multivariate procedures designed for use with a small number of variables (such as MANOVA and multivariate regression analysis) – many researchers have resorted to analysis strategies that consider some form of data reduction, such as cluster analysis and factor analysis (Alter et al., 2000; Quackenbush, 2001).

Although data reduction strategies have yielded important insights and have continually been refined, they do suffer from at least four problems. First, there are a myriad of different strategies for cluster analysis [such as hierarchical clustering (Eisen et al., 1998), *k*-means clustering (Tavazoie et al., 1999), self-organizing maps (Tamayo et al., 1999), etc.], and related strategies, making it difficult to know which approach might be the most appropriate for a given situation. Second, it is often difficult to determine, with some confidence, just how many clusters, eigenvalues, principal components, latent factors, etc., underlie or best represent any given data set. Third, the generalizability of the, e.g., clusters or principal components identified from a data set, as well as their ultimate biological meaning, is often in doubt. Lastly, many data reduction procedures focus on the initial “reduction” of the dimensions of the data into a few clusters, principal components, or latent factors, and do not necessarily provide a means for drawing probabilistic inferences about the relationships of the high-dimensional data to ancillary variables of interest which, in fact, may have motivated the study in the first place. Thus, for example, one may be interested in relating tumor gene expressions patterns gathered on a set of patients to their survival or other clinical outcomes. Although one could identify clusters of patients based on their tumor gene expression profiles and test to see if the patients in those clusters exhibited different survival rates, such approaches tend to be *ad hoc* and raise additional issues.

We have been developing an alternative and complementary data analysis approach to data reduction procedures that does not rely on – but could still exploit aspects of – data reduction strategies. This approach, termed Multivariate Distance Matrix Regression (MDMR) analysis, is rooted in traditional linear models and was first briefly proposed in the literature by McArdle and Anderson (2001) and Anderson (2001). MDMR provides a method for testing the association between a set of ancillary or “independent” variables, such as a clinical outcome in a tumor gene expression study, and high-dimensional data of the type produced by modern high-throughput biological assays. MDMR considers the data arising from a high-dimensional assay as providing a multivariate profile of each individual in the study. The similarity and differences in these profiles are then used to construct a distance or dissimilarity matrix whose elements are tested for association with ancillary (independent) variables of interest. Thus, MDMR is not unlike many data reduction strategies in that it requires a distance matrix. However, unlike data reduction strategies, MDMR tests the association between the elements of the distance or dissimilarity matrix directly with the ancillary variables and therefore does not require the problematic data reduction step. MDMR can be used with all the variables resulting from a high-throughput biological assay or some subset, making it a flexible and attractive tool for identifying meaningful patterns in high-dimensional data sets.

We have described applications of MDMR to actual biological data analysis settings involving genotype data (Wessel and Schork, 2006) and gene expression data (Zapala and Schork, 2006). However, to date there has not been a study investigating the properties of the MDMR procedure, including relevant test statistic distributions, the power of MDMR, and the robustness of the procedure. In the following, we examine the properties of the test statistics used in MDMR analysis in a wide variety of settings. We find that the MDMR test statistics and the procedure as a whole have some very desirable properties, such as an intuitive number of degrees of freedom for use in assessing the distribution of appropriate test statistics, an excellent test level accuracy, good power, and a flexibility that will make it an excellent adjunct or alternative to data reduction-based multivariate analysis strategies.

## Materials and Methods

We describe the MDMR analysis procedure by considering different aspects of its formulation and properties. We note that although graphical displays of distance matrices are not an essential ingredient of MDMR analysis, we include a discussion of graphical representations because they are used routinely in contexts for which MDMR analysis is appropriate.

### Computing a distance matrix

The formation of an appropriate distance (or dissimilarity) matrix is an essential ingredient in MDMR analysis. However, there are a large number of potential distance measures one could use to construct this matrix (Webb, 2002) and unfortunately there is very little published material that can be used to guide a researcher as to which distance measure is the most appropriate for a given situation. For example, although the Euclidean distance measure is used routinely in traditional cluster analysis settings, functions of the correlation coefficient are the most widely used distance measures in high-dimensional gene expression analyses (D’Haeseleer, 2005). We note that distance measures with either metric or non-metric properties can be used in the MDMR analyses (Gower and Krzanowski, 1999). Assuming that one has identified an appropriate distance measure, an *N* × *N* distance matrix is constructed. Let this distance matrix and its elements be denoted by *D* = *d_ij_* (*i*, *j* = 1, …, *N*) where *d_ij_* reflects the distance between profiles *i* and *j*.

### MDMR test statistic derivation

Once one has computed a distance matrix, *D*, the relationship between *M* additional factors (i.e., “ancillary,” “independent,” “predictor,” or “regressor” variables) collected on the individuals (e.g., diagnosis, age, gender, blood pressure level, etc.) and variation in the distances between and among the *N* individuals represented in *D* can be explored. Let *X* be an *N* × *M* matrix harboring information on the *M* factors which will be modeled as the independent or regressor variables whose relationships to the values in the distance matrix are of interest. Compute the standard projection matrix, *H* = *X*(*X*′*X*)^−1^*X*′, typically used to estimate coefficients relating predictor variables to outcome variables in multiple regression contexts. Next, compute the matrix $A=(a_{ij})=(-[1/2])d_{ij}^{2}$ and center this matrix using the transformation discussed by Gower (1966) and denote this matrix *G*:

(1) $$G=\left(I-\frac{1}{N}{\text{11}}^{\prime }\right)A\left(I-\frac{1}{N}{\text{11}}^{\prime }\right)$$

where **1** is an *N*-dimensional vector of 1’s. An *F*-statistic can be constructed to test the hypothesis that the *M* regressor variables have no relationship to variation in the distance or dissimilarity of the *N* subjects reflected in the *N* × *N* distance/dissimilarity matrix as (McArdle and Anderson, 2001):

(2) $$F=\frac{\text{tr}\left(HGH\right)}{\text{tr}\left[\left(I-H\right)G\left(I-H\right)\right]}$$

If the Euclidean distance is used to construct the distance matrix on a single quantitative variable (i.e., *P* = 1, as in a univariate analysis of that variable) and appropriate numerator and denominator degrees of freedom are accommodated in the test statistics, the *F*-statistic above is equivalent to the standard ANOVA *F*-statistic (McArdle and Anderson, 2001). The appropriate number and degrees of freedom to use in assessing significance of the test statistic in situations involving multiple variables (*P* > 1) and non-Euclidean distances measures is one of the main items to be explored in the studies described in the Section “Results” below.

### Collinearity

A fundamental problem with all multiple regression based analysis techniques is collinearity or strong dependencies (i.e., correlations) among the regressor variables. Collinearity can create problems in the computation of the projection matrix *H* = *X*(*X*′*X*)^−1*X*^′ as well as result in unstable parameter estimates. Although there are procedures that can be used to overcome this problem, such as ridge regression and principal components regression (Mason and Perreault, 1991), we have taken advantage of orthogonal-triangular decomposition (Gunst, 1983) to form the projection matrix and have found that this works well within the context of MDMR analysis.

### Permutation tests

The distributional properties of the *F*-statistic would be complicated to derive analytically for different non-Euclidean-based distance measures, especially when these distance measures are computed across more than one variable. Simulation-based tests, such as permutation tests, can then be used to assess statistical significance of the pseudo *F*-statistic as alternatives to the use of tests based on the asymptotic distribution of the *F*-statistic (Jockel, 1986; Edgington, 1995; Manly, 1997; Good, 2000). Permutation tests can be pursued by permuting the independent or predictor variables, recomputing the MDMR statistic, repeating this process, and tallying the number of times the statistics computed with the permuted data are larger than the statistic generated with the actual data. Despite the appeal of permutation tests, we have pursued an investigation of the utility of the *F*-distribution in assessing the significance of the proposed pseudo *F* test in contrast to permutation-based tests, as discussed in depth below. In addition, for large *N* permutation tests might be computationally inefficient with MDMR. We also note that the *M* regressor variables assessed in an MDMR analysis can be tested individually or in a step-wise manner (McArdle and Anderson, 2001; Zapala and Schork, 2006).

### Graphical display of similarity matrices

Distance matrices of the type to be used in MDMR analysis can be represented graphically in a number of ways and these graphical techniques can facilitate interpretation of the results of MDMR analysis. Two of the most widely used graphical representations include “heatmaps” and coded “trees” or dendrograms (Hughes et al., 2004; Kibbey and Calvet, 2005; Trooskens et al., 2005). Heatmaps simply color code the elements of a similarity matrix that is derived from a distance matrix, such that higher similarity values are represented as “hotter” or more red colors and lower similarity values are represented as “colder” or more blue colors. If the matrix is ordered such that individuals with similar values of one of the *M* potential regressor variables in an MDMR analysis are next to each other, then neighboring cells along the diagonal of the matrix (representing individuals with similar regressor values) will present patches of red, indicating a relationship between a regressor variable and similarity. Trees are constructed such that individuals with greater similarity (i.e., less distance) are placed next to each other (i.e., they are represented as adjacent branches of the tree) and less similar individuals are represented as branches some distance away from each other. By color coding the individual branches based on the values of a regressor variable possessed by the individuals they represent, one can see if there are patches of a certain color on neighboring branches, which would indicate that the regressor variable clusters along with similarity. Similarity matrices can be easily derived from distance matrices using appropriate transformations, such as dividing each entry in the distance matrix by the empirical or theoretical maximum distance and subtracting this value from 1.0.

### Cluster analyses involving distance matrices

Many forms of cluster analyses involve the use of distance matrices, such as hierarchical clustering techniques (Krzanowski, 1990). As noted in the Section “Introduction,” one particularly thorny issue in cluster analysis is the determination of the optimal or most representative number of clusters in a data set. The MDMR analysis technique has utility either as an alternative to cluster analysis or as a method for determining the optimal number of clusters. To determine an optimal number of clusters using MDMR, one could fit some number of clusters to a data set using a specific technique (such as *k*-means clustering; Webb, 2002), then assign individuals to specific clusters assuming this number of clusters and, using dummy codes for cluster membership, treat cluster membership as regressor variables in an MDMR analysis. One can then compare the test statistics resulting from the MDMR analyses for different number of clusters and choose as the optimal number of clusters that number of clusters for which the addition of clusters do not add significantly to the improvement in, e.g., percentage of variation explained, based on the MDMR analysis. Although our motivation for assessing the properties of the MDMR method is rooted in our belief that MDMR is an important alternative to cluster analysis, we have also considered studies that assess the utility of the MDMR as a way of determining the optimal number of clusters in a cluster analysis.

## Results

### Test level accuracy

The test level accuracy for the permutation test-derived *p*-values as a function of sample size was assessed with simulated data. Test level accuracy reflects how well the test controls the type I error rate. Thus, if a type I error rate of 0.05 is assumed in an analysis, a test with appropriate level accuracy would reject the null hypothesis 5% of the time. Hundred samples (*N* = 100) were generated each with 10 random variables (*P* = 10) following a standard normal distribution with a mean of 0 and a variance of 1. Fifty samples were assigned to a control group (0) and 50 samples were assigned to an experimental group (1). Thousand simulations were generated in this setting, which thus involved a single regressor variable (*M* = 1) representing group membership (i.e., coded as 0 = not in a specific group or 1 = in a specific group) that was not associated with the 10 variables used to construct the distance matrix. We reduced the sample size from 100 incrementally and performed additional simulation studies to explore the level accuracy of the test as a function of sample size. Table 1 describes the results and suggests that as the sample size decreases, the permutation test level accuracy declines, which is expected to occur.

**Table 1:** **Level accuracy of a permutation test as a function of decreasing sample size over 1000 simulations for a single dichotomous (categorical) predictor variable**.

(%)	*N* = 100	*N* = 50	*N* = 20	*N* = 10	*N* = 4
1	1.4	1.2	1.0	0.5	0.0
5	5.8	6.4	5.1	4.9	0.3
10	10.7	11.0	9.3	11.2	2.0
25	25.1	24.7	29.0	25.8	10.9
50	51.4	47.5	53.4	51.0	39.3
75	75.8	75.1	74.8	78.4	69.5

The level accuracy is slightly improved when continuous variables are considered as regressor variables. We generated 100 samples that had 10 random variables following a standard normal distribution with a mean of 0 and a variance of 1, as in the previous setting. A random variable with mean of 0 and variance of 1 was generated for each sample and used as a single continuous regressor variable (*M* = 1). Thousand simulations in this setting were conducted. Table 2 describes the results and suggests that permutation tests involving a single continuous regressor variable tend to have better level accuracy than those involving a single dichotomous regressor variable (compare Tables 1 and 2). We note that test level accuracy assuming different distance metrics was addressed in previously published work and suggests that different distance matrices do not have an appreciable effect on the behavior of permutation tests (Zapala and Schork, 2006). In addition, we have tested the level accuracy with bimodal distributions and log normal distributions (results available as Appendix) and obtain similar results to the normal distribution test level accuracy.

**Table 2:** **Level accuracy of permutations as a function of decreasing sample size over 1000 simulations for continuous variables**.

(%)	*N* = 100	*N* = 50	*N* = 20	*N* = 10	*N* = 4
1	1.4	1.5	1.2	1.6	0.0
5	5.5	5.4	5.4	5.7	3.5
10	10.3	11.2	11.1	12.2	7.3
25	24.0	26.7	25.0	24.7	21.2
50	46.6	51.3	51.3	50.7	48.1
75	72.6	74.7	76	74.9	73.5

### Comparison with *F*-statistic and *F*-distribution

**Figure 1:**
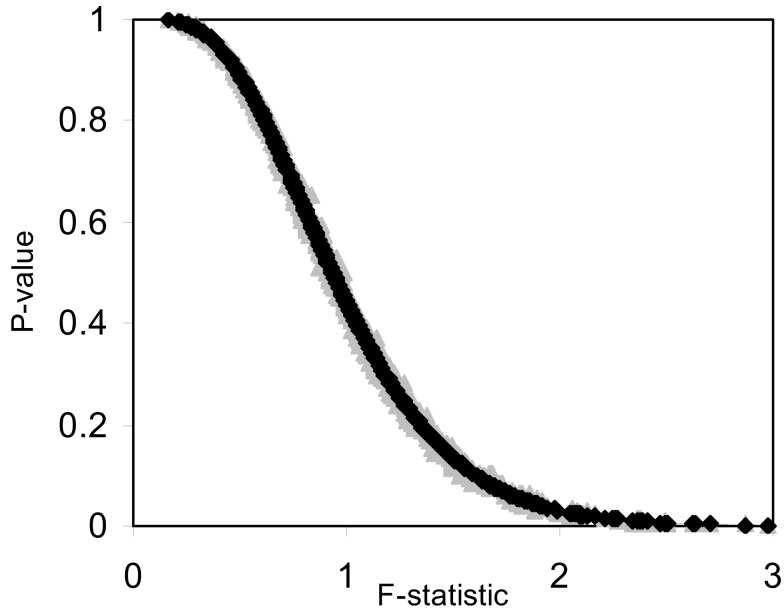
**Plot of permutation test-derived *p*-values as a function of the *F*-statistic in gray, the corresponding *p*-values derived from the *F*-distribution are overlaid in black for 100 samples and 10 random variables following a normal distribution with a mean of 0 and a variance of 1 simulated 1000 times**. Fifty samples were coded as control (0) and 50 samples were coded as experiment (1).

**Figure 2:**
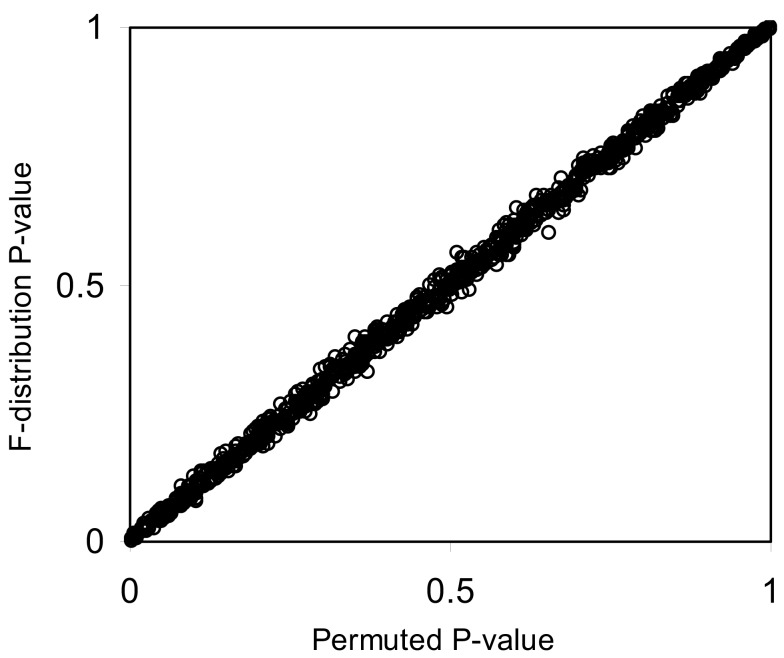
**Scatter plot of *p*-values from Figure 1 generated from permutation tests vs. those derived from the *F*-distribution (Pearson correlation coefficient = 0.99)**.

The pseudo *F*-statistic defined in Eq. 2 has a clear relationship to the *F*-distribution that is based on the number of quantitative variables that go into the construction of the distance matrix as well as the sample size. For a Euclidean-based distance matrix involving a single variable, the appropriate degrees of freedom are related to both the sample size and the number of variables used to create the distance matrix, as noted. This can be generalized such that if one has *N* subjects for which there are *P* quantitative variables that will be used to create the distance, the numerator, and denominator degrees of freedom for the pseudo *F*-statistic will be *P* and (*P* × *N*) – 2 respectively, which reduces to the appropriate degrees of freedom for the standard ANOVA. We expanded the simulation studies of the type discussed in Section “Test Level Accuracy” (i.e., 100 samples, 10 variables) to compare *p*-values resulting from permutation tests to those derived from the *F*-distribution with *P* and (*P* × *N*) – 2 degrees of freedom. Figures 1 and 2 provide two different ways of depicting the relationship between permutation-based *p*-values and the *F*-statistic-derived *p*-values and show a clear relationship between the pseudo *F*-statistic, the permutation test-derived *p*-values and the *F*-distribution derived *p*-values. This suggests that the *F*-statistic provides a reliable and level-accurate hypothesis testing for MDMR analyses in certain settings.

**Figure 3:**
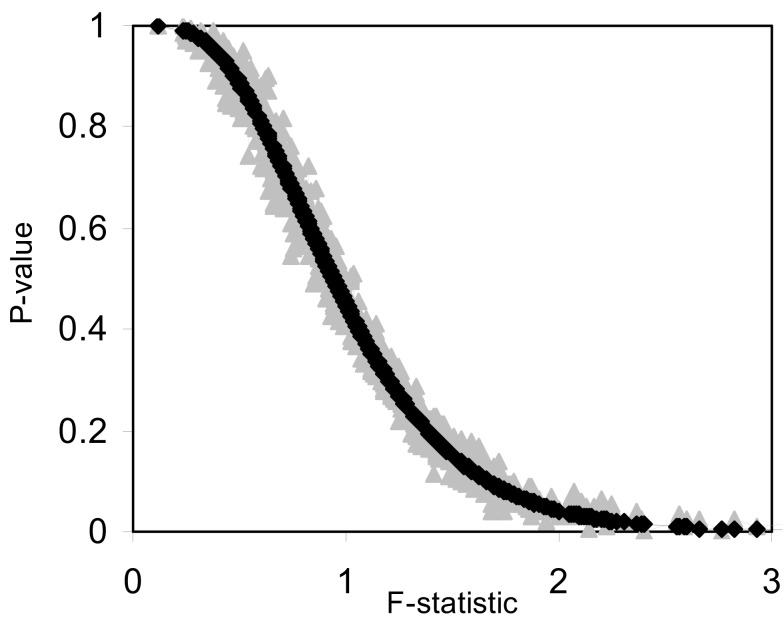
**Plot of permutation test-derived *p*-values as a function of the *F*-statistic in gray, the corresponding *p*-values derived from the *F*-distribution are overlaid in black for 10 samples (*N* = 10) and 10 random variables (*P* = 10) following a normal distribution with a mean of 0 and a variance of 1 simulated 1000 times**. Five samples were coded as control (0) and five samples were coded as experiment (1).

We also investigated the correspondence of the permutation test-derived *p*-values and the *F*-distribution derived *p*-values for small sample sizes. Figure 3 and Table 3 provide the results of these investigations and clearly show that permutation test and *F*-distribution derived *p*-values do not agree well with samples of size 10 as opposed to 100 (Figure 1). Thus, the size of the matrix, which is related to the number of subjects, affects the accuracy of the permutation test and related *F*-distribution-based test.

**Table 3:** **Level accuracy of *F*-distribution *p*-values as a function of decreasing sample size over 1000 simulations for a single dichotomous (categorical) predictor variable**.

(%)	*N* = 100	*N* = 50	*N* = 20	*N* = 10	*N* = 4
1	1.5	0.8	1.5	1.3	2.3
5	5.5	6.2	5.2	5.7	8.0
10	10.5	11.3	10.4	11.0	12.8
25	25.2	24.6	28.9	25.9	26.6
50	51.5	46.8	53.3	52.1	52.4
75	76.2	74.9	75.0	77.6	75.1

**Figure 4:**
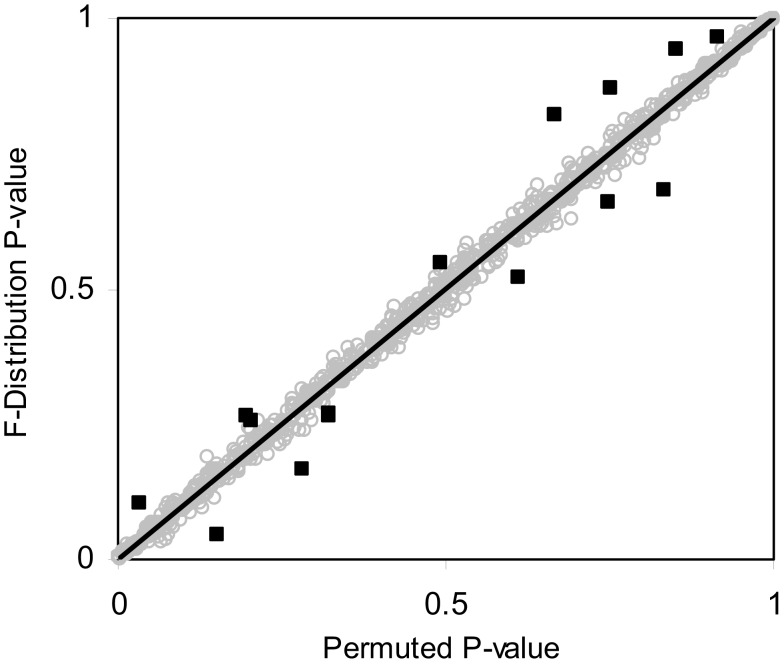
**Scatter plot of *p*-values obtained from the *F*-distribution vs. permutation tests for random samples sizes varying between 4 and 100 (i.e., 4 ≤ *N* ≤ 100) and random variables size from 1 to 100 (i.e., 1 ≤ *P* ≤ 100) with a single continuous regressor variable (*M* = 1) simulated 1000 times**. Outlying observations represented as black squares lying away from the trend line have sample sizes less than or equal to eight.

Table 3 suggests that for samples of size 10 or less the accuracy of the *F*-distribution based *p*-values suffer; however, it is considerably more accurate than the permutation test-derived *p*-values (compare Table 1). Figure 4 provides a scatter plot comparing *p*-values obtained from permutation tests vs. *p*-values obtained from the *F*-distribution for samples with sizes between 4 and 100 samples and a random number of variables ranging from 1 to 100 for MDMR analysis settings involving a single continuous regressor variable. Figure 4 clearly shows that smaller sample sizes (*N* ≤ 8) show marked differences between the permutation test-derived *p*-values and the *F*-distribution derived *p*-values.

### Power

**Figure 5:**
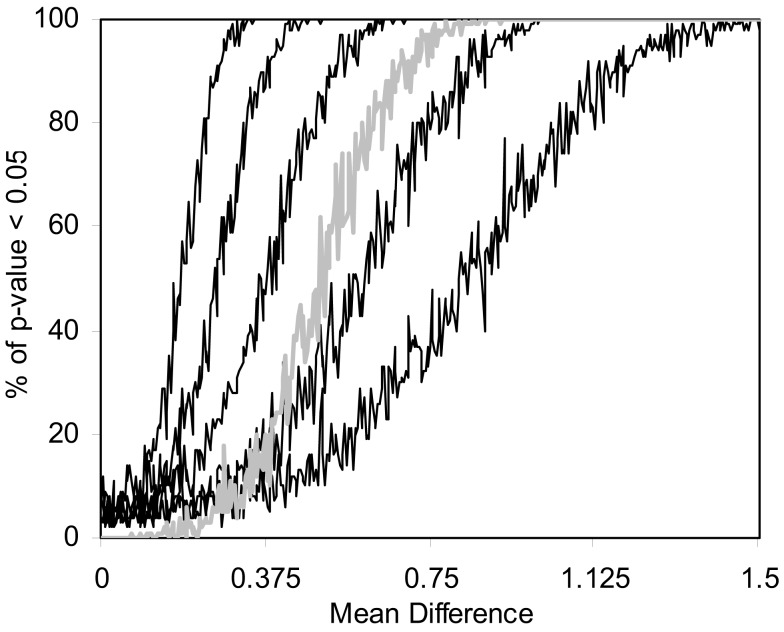
**Power of the MDMR procedure as a function of signal-to-noise ratio obtained from 1000 simulated data sets for a wide variety of settings**. Simulated data for 30 (*N* = 30) samples and 100 variables (*P* = 100) were generated with 15 samples assigned to a control group (independent variable = 0) and 15 samples assigned to an experimental group (independent variable = 1). Random data in the control group were generated as standard normal variates with a mean of 0 and variance 1. Random data in the experimental group were generated as standard normal variates with variance = 1 and means that took on values of 0–1.5 in increments of 0.001. The power of the permutation-based statistical test is presented. We generated different simulated data sets for which 100, 50, 25, 10, or 5% of the variables used in the construction of the distance matrix had means adjusted from 0 (in the appropriate increments) in the experimental group. The gray line shows the power of a Bonferroni corrected *P*-value for the Student’s *t*-tests performed on each of the 100 variables in univariate *t*-tests which were corrected for the hundred statistical tests pursued.

**Figure 6:**
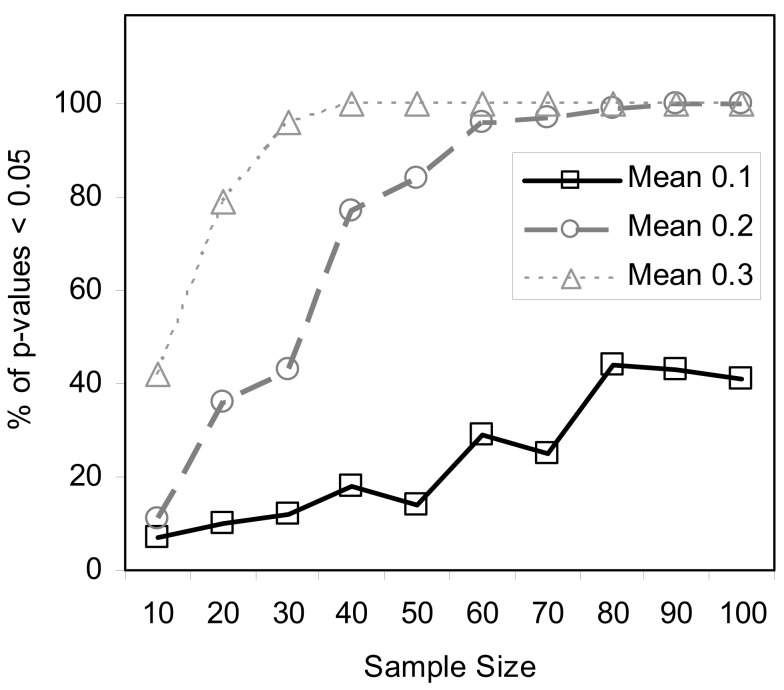
**Power of the MDMR procedure as a function of increasing sample size**. Half of the samples for each sample size were assigned to a control (coded as 0) and half to an experimental group (coded as 1). For each sample 100 random variables were generated following a normal distribution with a mean of 0 and a variance of 1 for the control group and an assigned mean difference of 0.1, 0.2, or 0.3 and a variance of 1 for the experimental group.

We also pursued simulation studies to explore the power of the MDMR procedure in a variety of settings. Our initial power studies considered 30 samples (*N* = 30) with 100 variables (*P* = 100), where these 100 variables were generated as standard normal variates. We then added a value, in increments of 0.001, to the means of the variables for 15 of the 30 subjects and tested the association between a single dichotomous categorical regressor variable (coded as 0 for the first 15 subjects and 1 for the second 15 subjects) and the distance matrix computed from the 100 variables for each subject via the Euclidean distance measure. Figure 5 displays the results for settings in which different proportions of the 100 variables had increments of 0.001 added to them for the second 15 subjects. As can be seen, when all the variables have their means adjusted for the second 15 subjects, MDMR can detect a mean difference of 0.24 standard deviation units 80% of the time, whereas Bonferroni corrected Student’s *t*-tests pursued on each of the *P* variables individually can detect a mean difference in one of the variables of 0.62 standard deviation units 80% of the time. We also pursued power studies where the variables followed a bimodal distribution (and found that power is the same as a single mode normal distribution), log normal distributions (using a mean value of 0.17) as well as multivariate normal distributions (using a correlation among the variables of 0.06). These simulation studies (available as Appendix) demonstrated that the MDMR procedure has similar power to detect differences in these settings and thus suggests that the MDMR procedure is robust and can detect subtle differences in groups over a range of conditions. We also considered the power of the MDMR procedure as a function of sample size. Figure 6 depicts the results for increasing sample size assuming different mean differences between the 100 normally distributed variables in two groups. It can be seen that samples sizes greater than 40 are able to identify mean differences of 0.2 or greater 80% of the time.

**Figure 7:**
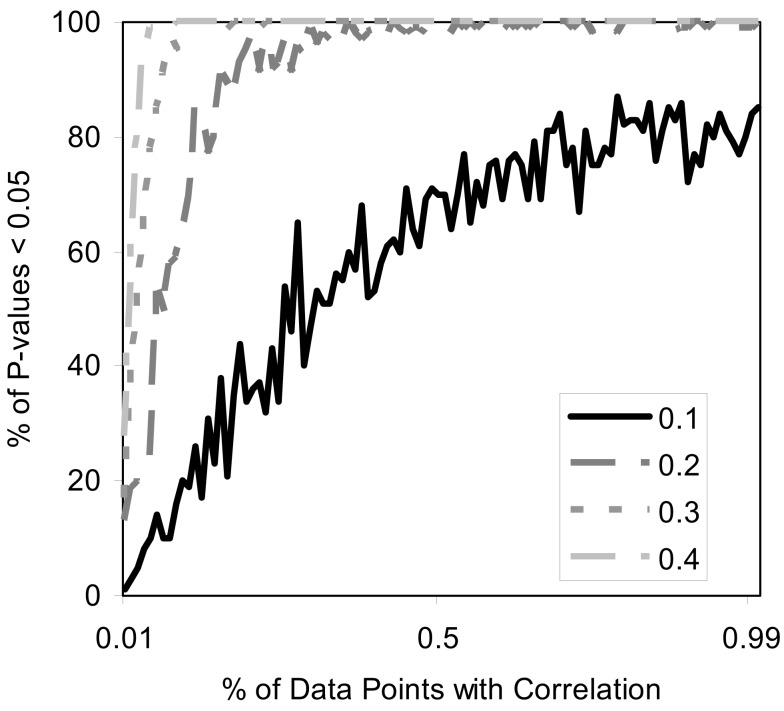
**Power of the proposed MDMR procedure as a function of the correlation of continuous regressor variables for a samples size of *N* = 100 with *P* = 100 variables**. The *x*-axis displays the percentage of variables that have a correlation to the regressor variable. Four different correlation strengths are shown ranging from 0.1 to 0.4. *P* = 100 random variables were generated following a normal distribution with a mean of 0 and a variance of 1.

Finally, we studied the power of the MDMR procedure with continuous regressor variables. We induced relationships between the continuous regressor variables and the *P* variables assigned to each subject used to construct the matrix by assuming that the regressor variable was correlated at some level with either each of these *P* = 100 variables or some fraction of them. Figure 7 depicts the results and shows that the MDMR procedure can identify relationships among data points when 15% of variables are correlated with the regressor variables at a strength of 0.2. Higher correlations allow a smaller percentage of the variables to be correlated with the regressor before the relationships are detectable with MDMR. For situations in which one may have multiple variables (i.e., *P* > 1) we note that MDMR is flexible enough to be used in a univariate manner to analyze each variable independently (*P* = 1) and identify a subset of variables for which the regressor has the strongest association with variation in the distance matrix as a whole. MDMR can then be used in a multivariate manner to determine if the overall effect of the regressor is increased by looking at these data points together. In this way, MDMR can reduce the possibility of over-fitting data and identify optimal subsets of variables related to a set of additional factors or regressor variables.

### Determining the optimal number of groups in a cluster analysis

**Figure 8:**
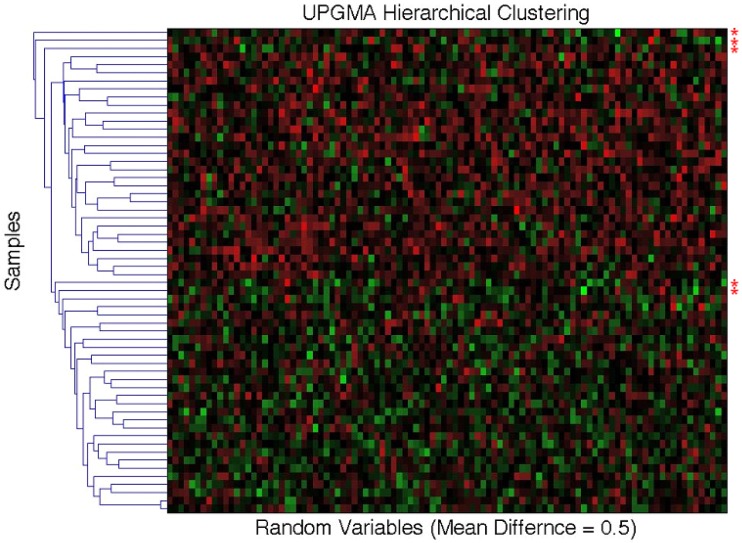
**Comparison of the UPGMA hierarchical cluster algorithm to the matrix regression technique**. Simulated data for *N* = 60 samples and *P* = 100 variables were generated with 30 samples assigned to the control group (independent variable = 0) and 30 samples assigned to the experimental group (independent variable = 1). Random data in the control group were generated as standard normal variates with a mean of 0 and variance of 1. At mean differences below 0.75, hierarchical clustering using the unweighted average distance (UPGMA) does not clearly differentiate two groups with different means. Shown above are five clusters for what visually appears to be two groups. The red asterisks (*) signify simulated data that has been misclassified. Two samples whose means were at 0.5 were grouped with samples whose means where 0 (bottom two asterisks). The matrix regression technique shows that the correct grouping of two separate groups gives the highest *F*-statistic of 5.32, while the UPGMA clustering technique of five distinct groups only provides an *F*-statistic of 5.28.

As noted throughout this paper, MDMR analysis provides an alternative to many standard multivariate analysis techniques, including cluster analysis techniques. Cluster analysis has been a common strategy used to identify patterns in high-dimensional, *P* ≫ *N*, data sets. However, given the vast array of cluster analysis strategies that have been proposed, it is often unclear which cluster analysis method is most appropriate for a particular setting. Furthermore, cluster analysis techniques rarely provide formal statistical tests to relate predictor or regressor variables to the clusters arising from an analysis and often provide ambiguous answers to questions concerning the optimal number of clusters present in a dataset. We have compared the common UPGMA (Unweighted Pair Group Method with Arithmetic mean) hierarchical clustering technique to the MDMR procedure in a single analysis setting to showcase the potential MDMR has to complement cluster analysis strategies. We generated data for two groups of subjects of size *N* = 30, where each subject was assigned *P* = 100 variables as standard normal variates. Then, for the second group of subjects, we added a value to the means of each of the 100 variables. We then pursued cluster analysis on the resulting data sets and tested to see if the number of groups identified from the cluster analysis was consistent with the number of groups producing the highest and most significant (in terms of *P*-value) *F*-statistic from the MDMR analysis (as described in section), where predictor variables were created reflecting cluster analysis-derived group membership and tested for association with the distance matrix. We found that for mean differences less than or equal to 0.75 standard deviation units, UPGMA clustering has difficulty identifying two distinct groups for a sample size of 60. MDMR was shown to accurately identify mean differences of greater than 0.2 for a sample size of 60 (see Figure 6). Figure 8 provides an example of the phenomenon where UPGMA clustering suggested that there were five groups with some misclassified observations, although the MDMR analysis suggested two groups were the most likely. Thus, MDMR analysis can be used to create tests for the optimal number of groups in a cluster analysis. We are exploring this theme further in additional work.

## Discussion

Our studies suggest that the MDMR analysis procedure has exceptional promise as an adjunct or alternative to standard multivariate analysis methods for use with modern high-throughput biological assays. The MDMR procedure is ideally suited for settings in which *P* ≫ *N*, and where a researcher is ultimately interested in analyzing multivariate data collected on a group of individuals as though those data were providing multivariate “profiles” of the individuals, rather than as data reflecting a distinct set of variables requiring independent attention. Such settings are the rule, rather than the exception, in many modern biological experiments. For example, gene expression studies are typically pursued to address questions about the “state” of a cell or tissue type at a particular time or after a particular intervention. Although there is great interest in finding particular genes whose expression levels differ the most between times or interventions, there is also great interest in determining if the overall expression profiles of the genes have been altered or if particular groups of genes, defined by biochemical pathways or networks, have been changed. By constructing multivariate gene expression profiles of all (or subsets) of the genes whose similarities and differences can be interrogated, one can test hypotheses about the overall state of the cell or tissue. For example, we have previously shown that genes involved in Pharm-GKB derived ACE-inhibitor pathway show altered multivariate gene expression patterns in the kidneys of patients with renal disease which is consistent with their levels of tubular atrophy/interstitial fibrosis (Zapala and Schork, 2006). This analysis formally tested a well-established hypothesis, that the renin-angiotensin-aldosterone system (RAAS) plays a role in renal fibrosis (Lewis et al., 2001). This type of hypothesis could not have been tested using traditional univariate or clustering approaches. We emphasize, however, that this type of analysis is in no way limited to this particular pathway-based hypothesis, but rather can be extended to other sets of genes.

As another example, consider modern high-throughput DNA sequence data. Such data are often generated to address questions about the evolutionary relationships between species or the divergence of individuals within a species based on events such as migration, isolation, drift, and/or phenotypic divergence (Wessel and Schork, 2006; Nievergelt et al., 2007). A fundamental step in the analysis of DNA sequence data to address such questions is the derivation and use of a measure of DNA sequence similarity (Clark, 2006; Phillips, 2006). Once one has quantified just how similar or different various DNA sequences are, hypotheses about the factors that may be associated with the differences can be framed. MDMR analysis would be an ideal tool for testing these hypotheses, especially since one would not likely be interested in testing hypotheses about differences at each nucleotide, but rather the DNA sequence as a whole or a profile.

Our studies also show that the properties of test statistics for pursuing MDMR analysis are quite good, in that they are well-behaved, exhibit an excellent level accuracy, and have good power to detect a wide-range of multivariate phenomena. In addition, by confirming that the *F*-statistic used to test associations within the MDMR framework follows an *F*-distribution with an intuitive number of degrees of freedom, there is a computationally efficient alternative to permutation-based tests. This computational efficiency can be of great value if MDMR analyses are to be pursued in settings where repeated tests are to be performed, such as in testing associations between hundreds of thousands of DNA sequence variations and multivariate phenotypes within a genome-wide association study (GWAS).

There are a number of issues with MDMR analysis that need further attention. For example, the choice of an appropriate distance measure may be problematic. Although our experience suggests that different distance measures provide roughly the same inferences (Zapala and Schork, 2006), greater research into this issue should be pursued. In addition, the handling of missing data in both the construction of the distance matrix and in relating the regressor variables to the variation in the distance matrix is problematic. Handling missing data in the construction of the distance matrix may not be a huge problem if, for any pair of individuals in the sample *P* is large and they are only missing a few value between them. In this case, one could compute the distance measure with only the non-missing values. However, studies investigating the “critical level” of missing data that can be tolerated in this setting are needed.

What would be of greatest interest, however, is a comparison of MDMR analysis with other analysis methods that could be applied to similar types of data sets. For example, for small *P* in settings involving group comparisons, one could compare MDMR with standard MANOVA or multivariate regression analyses (as done, for example, by Waters and Cohen, 2006). More interesting comparisons might involve MDMR analyses in settings where *P* is large and cluster analysis, principal components, and related data reduction analysis techniques might be appropriate. Regardless of the outcomes of these proposed studies, MDMR analysis has a place in multivariate analysis as one of the few approaches to directly relate variation in a large set of variables to a set of potential explanatory variables.

The source code for this statistical method is written in Python and is freely available at the Biopython script central page^1^ and is being incorporated into the Biopython library. Also, the source code and a user friendly web application are available on the Schork Laboratory website^2^ Implementations of the MDMR technique are also available in R^3^.

## Conflict of Interest Statement

The authors declare that the research was conducted in the absence of any commercial or financial relationships that could be construed as a potential conflict of interest.

## Figures and Tables

**Figure A1:**
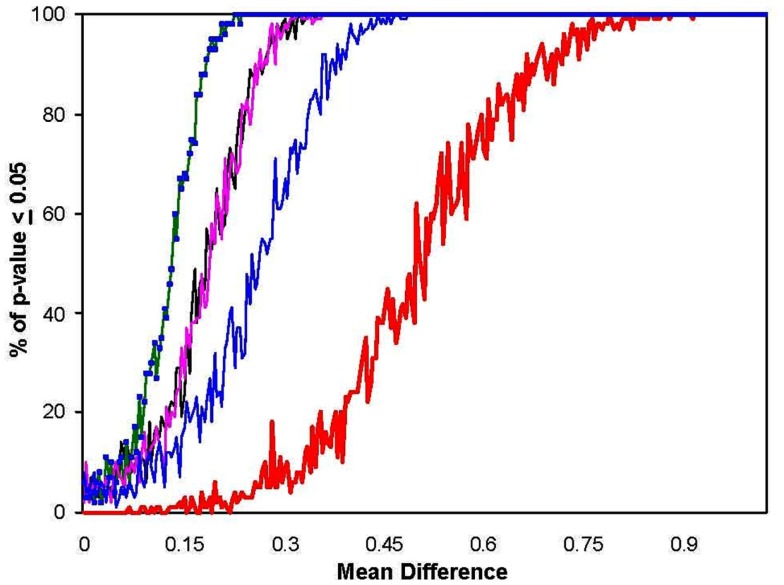
**Power of the MDMR procedure as a function of non-normal population distributions**. The black line shows power as calculated before for two populations with normal distributions. The green line displays power for populations with log normal distributions. The pink line shows power for power for populations with bimodal distributions (equivalent for a normal distribution with 100% of the data having means altered) and the blue line shows power when only one mode of a bimodal population is different (equivalent for a normal distribution with 50% of the data having means altered). The red line shows the power of a Bonferroni corrected *p*-value for the Student’s *t*-tests performed on each of the 100 variables in univariate *t*-tests which were corrected for the 100 statistical tests pursued.

**Figure A2:**
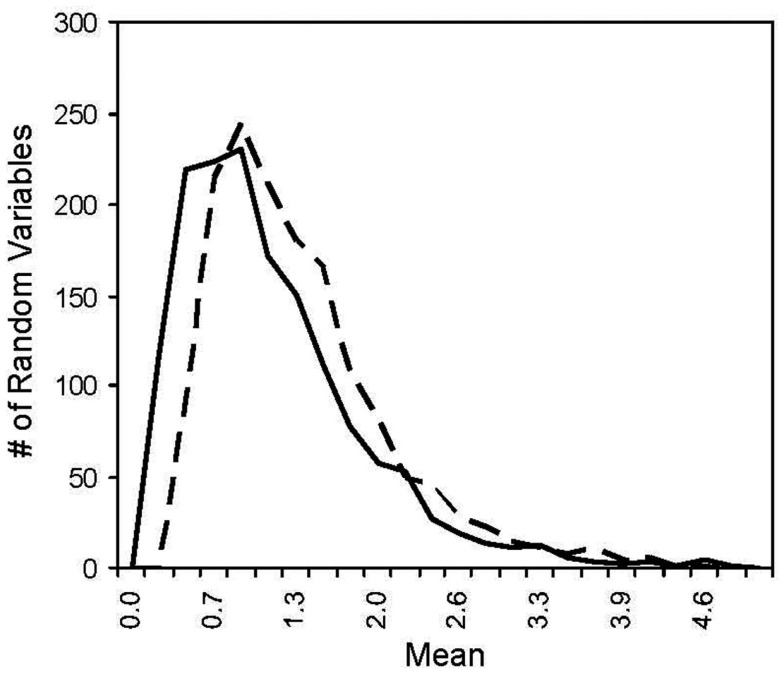
**Histogram of two log normal distribution**. The solid line has a mean of 1 and the dotted line has a mean of 1.225 where the difference in the means yields ~100% power for MDMR with a two log normal population distributions.

**Figure A3:**
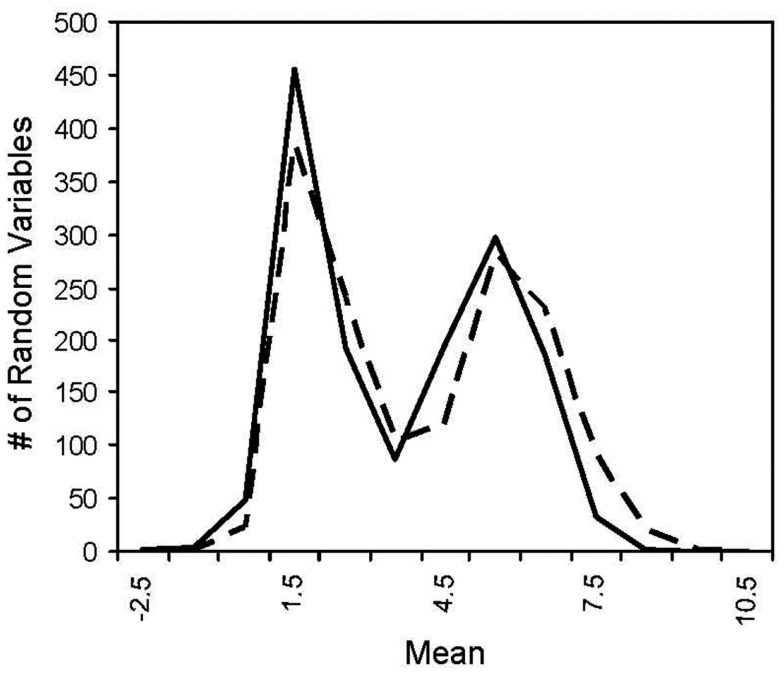
**Histogram of two bimodal distributions**. The solid line has two modes with a mean of 1 and a mean of 4 and the dotted line has two modes with a mean of 1.36 and a mean of 4.36 where the difference in the distributions yields ~100% power for MDMR.

## Footnotes

^1^http://biopython.org/wiki/Scriptcentral

^2^http://polymorphism.scripps.edu/~cabney/cgi-bin/mmr.cgi

^3^https://bcrc.bio.umass.edu/biometry/images/8/85/Vegan.pdf

## Appendix

**Table A1:** **Level accuracy of permutations as a function of decreasing sample size over 1000 Simulations for log normal distribution**.

(%)	*N* = 100	*N* = 50	*N* = 20	*N* = 10	*N* = 4
1	1	1.5	1	1	0
5	4.5	4.9	4.4	4.6	0.5
10	8.5	10.5	9	9.7	2.5
25	25.5	25	23.1	24.9	10.2
50	49.8	50.2	49.4	52.5	38.7
75	74.9	73.5	75.7	76.5	69.2

**Table A2:** **Level accuracy of permutations as a function of decreasing sample size over 1000 Simulations for bimodal distribution**.

(%)	*N* = 100	*N* = 50	*N* = 20	*N* = 10	*N* = 4
1	0.9	0.9	1.5	0.4	0
5	4.7	4.7	5.6	5.1	0.2
10	10.4	10	11.1	10.5	2
25	27.2	25.7	26.1	24.8	9.3
50	52.3	48.5	51.4	50.7	39.6
75	76.7	76.2	74.2	75.1	67.8
